# Spatial and Temporal Comparison of Perceived Risks and Confirmed Cases of Lyme Disease: An Exploratory Study of Google Trends

**DOI:** 10.3389/fpubh.2020.00395

**Published:** 2020-08-14

**Authors:** Dohyeong Kim, Sarah Maxwell, Quang Le

**Affiliations:** ^1^School of Economic, Political and Policy Sciences, University of Texas at Dallas, Richardson, TX, United States; ^2^Department of Bioengineering, University of Texas at Dallas, Richardson, TX, United States

**Keywords:** lyme disease, Google Trends, spatial, temporal, non-specific symptoms

## Abstract

Non-specific symptoms in later stages of Lyme disease (LD) may mimic a variety of autoimmune, viral, or complex diseases. Patients lacking erythema migrans or who test negative under CDC guidelines, but suspect LD may search online symptoms in vein. As a result, patients with lingering and undiagnosed symptoms turn to alternative lab tests. This study addresses patient's perceived illness in relation to CDC surveillance data. Extending the literature beyond basic searches for symptoms or disease terms, this study examines spatiotemporal dynamics among symptom, disease, and unconventional lab test searches on Google Trends, in compared with CDC confirmed cases of LD. The search terms used for the Google Trends analysis between 2011 and 2015 include: (1) “lyme” and “lyme disease” for disease, (2) “tick bite,” “bone pain,” “stiff neck,” “circular rash,” and “brain fog” for symptoms, and (3) “IGENEX” for the alternative lab test. Spatial and temporal analyses illustrate noticeable similar patterns between the search frequency and the actual LD incidence. Beyond basic searches for symptoms or disease terms, we demonstrate the improved utility of Google Trends analysis in discovering spatial and temporal patterns of perceived LD and comparing with the reported LD cases. The public health and medical communities benefit from this research through improved knowledge of undiagnosed patients who are searching for alternative labs to explain lingering symptoms. This study validates the need for further research into Google Trends data and surveillance protocols of diseases characterized by non-specific symptoms, prompting patients to “self-diagnose.”

## Introduction

Lyme Disease (LD), a tick-borne and multi-systemic infection caused by Borrelia burgdorferi sensu lato, is the most common vector borne illness in the United States, with an estimated 240,000–440,000 new cases each year (1, 2). The Centers for Disease Control (CDC) reports that within days to months following untreated exposure to LD, symptoms such as severe headache, nerve pain, dizziness, shortness of breath, additional rashes, and problems with short-term memory or brain fog may be present in a patient (3). However, non-specific symptoms of LD may also manifest in later stages due to a variety of autoimmune, viral, or complex diseases. Given the incidence of LD and presence of non-specific symptoms on which to clinically diagnose, it is believed that at least 3 million LD lab tests are ordered in the U.S. each year (4, 5).

Testing for LD follows the CDC's 2-tiered serological guidelines (6–8), yet variability and subjectivity are reported with the CDC-recommended protocol (6, 7, 9, 10). Clinical diagnoses of LD may be made in patients with erythema migrans (EM) (6), and rashes and fungal infections are known to be commonly mistaken for EM (2, 11). CDC's testing presents different levels of significance in accordance with symptom presentation, EM presence, or later-stage disease manifestation (12). LD testing may lack a positive predictive value (4, 13), primarily when testing is carried out in areas of low disease incidence (14) or in cases of early LD infection (11–13). In many situations, patients with EM find themselves with a variety of symptoms that present clinically as LD but fail to positively meet two-tier serologic indicators (4). Physicians and scientists thus face inconsistent indicators regarding LD incidence and potential diffusion in the United States.

Over time, patients with LD or other diseases become increasingly upset by persistent symptoms such as fatigue, flu-like symptoms, expensive, and repeated medical tests, and a perceived medical system that seems unable or uninterested in securing a diagnosis (4). If undiagnosed and untreated, patients with persistent symptoms may become concerned or alarmed, and go in search of alternative help. Given the difficulty in diagnosing LD in both acute and late stages (15), clinicians and patients in many cases rely on specialized testing (4, 6, 15). Patients who test negative under CDC guidelines but suspect LD may search online symptoms in vain, and turn to alternative lab tests, often from specialty and commercial labs. “Lyme specialty laboratories” (6) have emerged in the commercial marketplace, as have numerous “unorthodox” and “alternative therapies” (14, 16). Specialty labs are reported to have low specificity, indicating that up to 57% of the LD specialty labs result in false positives for LD (8), or are noted to present with potentially false positive results (4, 17, 18). Clinical cases also appear with non-LD testing, such as false-positive Epstein-Barr virus (EBV) serologies in LD patients (19). IGeneX is a specialty lab developed by a physician who was encountering patients with clinical symptoms of LD, but had negative two-tier testing. Test kits can be ordered by patient or physician, potentially leading to patient searches for the perceived appropriate diagnostic path. IGeneX tests are generally not ordered by mainstream physicians, pointing to the patient seeking further information or potential “diagnosis” of their symptoms.

Google Trends is a commonly employed tool in public health (20). Recent literature attempts to link infectious disease with Google Trends searches primarily for surveillance or descriptive purposes (21–24). To date, all Google Trends infectious disease studies employ search terms that either use the name of the disease or their symptoms, such as gonorrhea (25), HIV/AIDS (26), norovirus (27), dengue (28), influenza (29), urinary tract infection (30), and flu (31). These studies show promise in temporal alerts to infectious disease and coincide/correlate with endemic or highly-affected areas (32). While the symptoms of these diseases are relatively distinct and specific, various non-specific symptoms have been reported for LD, as described above. Limited scholarship is available on the use of Google Trends to estimate LD risks, and their search terms for symptoms are restricted to “tick bite” or “cough” (33, 34). Moreover, Google Trends studies have not explored lab test searches for infectious disease, including LD.

In both infectious and non-infectious disease studies, Google Trends is offered as a novel tool for understanding spatial or temporal patterns of diseases and risks. It can track disease employing basic pattern analyses such as internet search volume or basic seasonal patterns, but has a potential to be expanded to discover geospatial or spatiotemporal patterns. Although some recent studies suggest spatial and temporal applications of Google Trends in tracking non-infectious disease such as kidney stones or diabetes (35) and infectious disease such as cholera or malaria (36), the empirical literature is still at infancy, particularly for LD. Those studying the spatial dimensions of infectious disease typically rely on basic search terms for disease names or symptoms only. Given the complex nature of testing, coupled with non-specific symptoms related to LD, patients take it upon themselves to search for testing that is suggestive of underlying disease, inflammation, or chronic illness. Google Trends shows promise in determining types of tests suspected LD patients are exploring, comparing spatial patterns of commonly-used labs with CDC-confirmed LD cases, and assessing the efficacy of searches for specialty labs in LD endemic areas.

In this study, we extend previous literature in an effort to evaluate Google Trends data to better understand the dynamics of LD cases vs. LD concerns using a comprehensive search process. We use Google Trend search data to track LD both spatially and temporally in comparative perspective to non-specific symptoms and lab tests with possible *or perceived* sensitivity to LD infection, and compare the patterns with CDC confirmed cases of LD. We focus specifically on one commercial test that are prominent in the LD patient advocacy communities, IGeneX. By comparing patients in search of unconventional lab tests across multiple search indicators in addition to those who tested CDC-positive, we examine dynamics among symptom, disease, and unconventional lab tests, highlighting spatial and temporal mismatch between various search trends and actual disease incidence. Beyond basic searches for symptoms or disease terms, we demonstrate the improved utility of Google Trends analysis in discovering and comparing spatial and temporal patterns of LD cases vs. perceived risks.

## Methods

The number of confirmed LD cases by States from 2011 through 2015 was collected from the annual summary of Notifiable Diseases published by the CDC (37). Individual years were aggregated to obtain the overall number of confirmed cases through this period. Google Trends outcomes were extracted for a series of search terms in the United States during the years between 2011 and 2015. Google Trends shows search frequency by attributing the value of 100 to the maximum number of weekly hit and attributing to all other weeks their relative value as a percentage of the maximum number of hits. When two search terms are compared, each week is given 2 values, one for each term showing their relative number of hits. We take the sum of these two values for each week, which is considered the weekly search frequency. As for spatial outcomes of Google Trends with multiple search terms, the highest number of searches overall for one term in one state is assigned the value of 100 and all other items are given a value to represent their relative frequency compared to the maximum. We take the sum of search frequencies in all search terms for each state and graph that data over an US map.

Combining the data, we first examined temporal/seasonal (monthly) patterns of searching trends for (1) “lyme” and “lyme disease” for disease name, (2) “tick bite,” “bone pain,” “stiff neck,” “circular rash,” and “brain fog” for symptoms, and (3) “IGeneX” for the alternative lab test, and compare those with monthly trends of the CDC-confirmed Lyme Disease cases. Since Google Trends only returns exact matches, a number of different variations of search terms (i.e., chronic lyme disease) were also tested for robustness of the results, including other kinds of symptoms reported in the literature. We then compared spatial trends of all the items above by drawing state-level maps for the aggregated number of CDC-confirmed LD cases and the three Google Trends search frequency data from 2011 through 2015. We also compared county-level spatial variations within the state of Texas, only between the confirmed cases and disease name search outcomes. For all mapping schemes, the relative number of cases was represented using a grayscale with quantile break. The darker the state or county, the higher number of aggregated cases or searches.

## Results

### Temporal Trends

**Figure 1:**
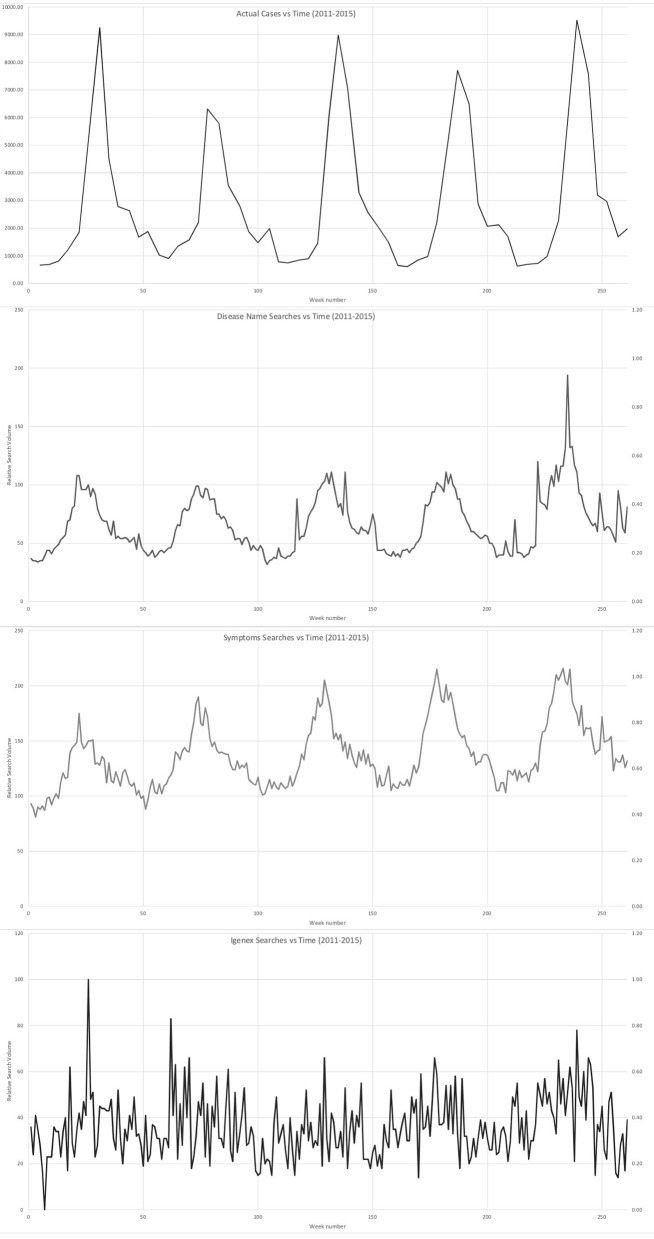
Temporal comparison of (1) CDC-confirmed LD cases with the Google Trends results for (2) disease name, (3) symptoms, and IGENEX (2011–2015).

Figure 1 compares the monthly trends of the CDC-confirmed LD cases with the Google Trends results for disease name, symptoms, and IGeneX for the years between 2011 and 2015. All four graphs clearly show seasonality, peaked during summer and fall. However, it seems evident that the peaks for the search patterns, both disease name and symptoms, precede to some extent those for the actual cases (June and July) (38). Such time lag between predicted and confirmed results corresponds to the previous literature on Google Trends (39). The pattern of seasonality found in symptom searches is mostly based on only one search word “tick bite” and the other four search terms for symptoms do not present any noticeable level of seasonality. Besides seasonality, the figures also compare a long-term trend over the 5 years; the number of confirmed LD cases remained the same while the search trends for disease names has gradually increased over time. The last figure for the search outcome for “IGeneX” also shows seasonality but with a lot of fluctuations, mostly due to small case numbers. It is believed that these figures support the argument that patients who had symptoms known for LD but negative test results under CDC guidelines tend to search online for alternative help, particularly lab tests from specialty and commercial labs such as IGeneX.

### Spatial Trends

**Figure 2:**
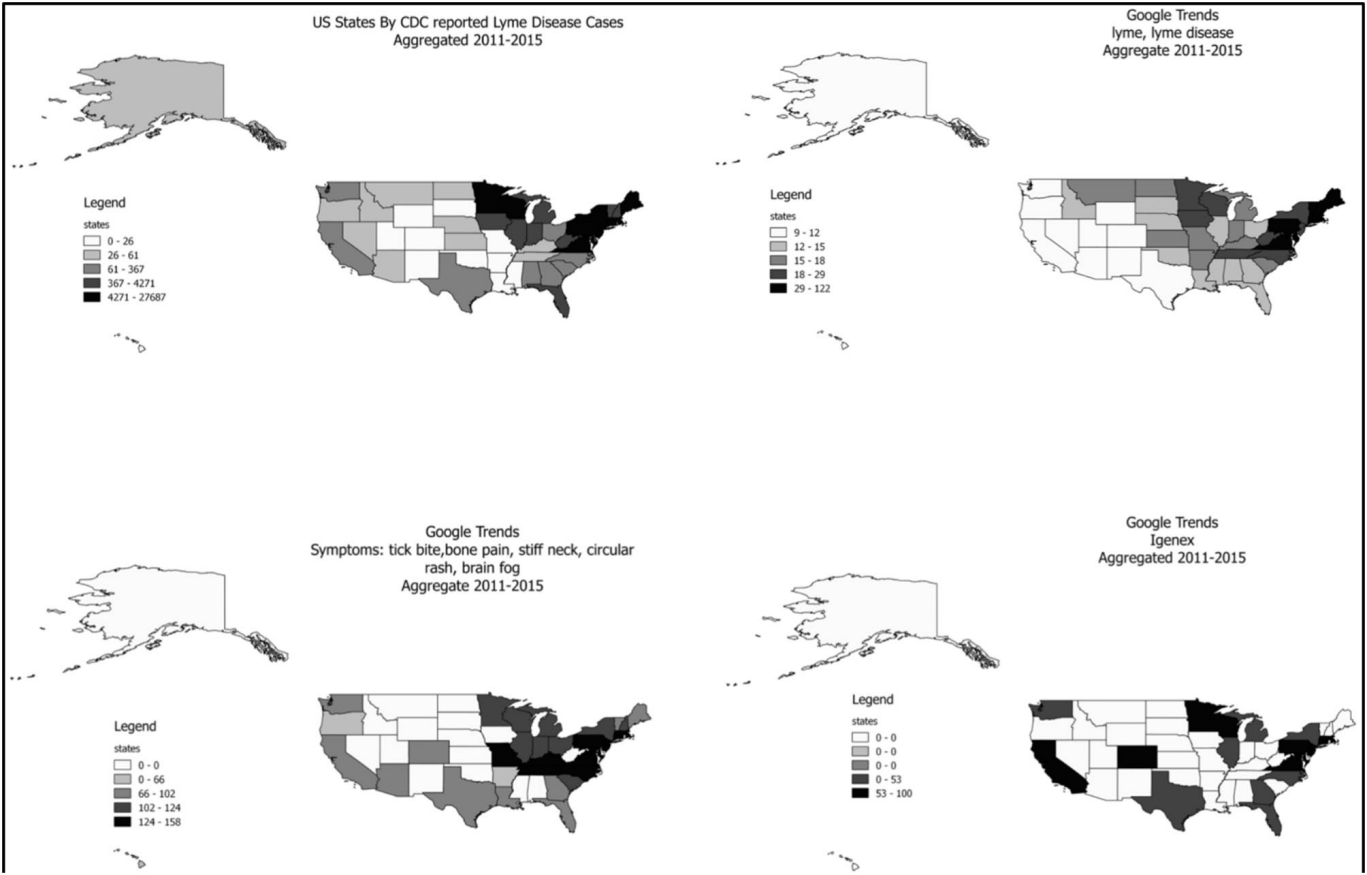
State-level spatial distribution of CDC-confirmed LD cases and Google Trends results for disease name, symptoms, and IGENEX.

Figure 2 illustrates state-level spatial variation of CDC-confirmed LD cases (top left) and Google Trends results for disease name, symptoms, and IGeneX. There seems a discrepancy between spatial pattern of the confirmed LD cases and various search trends. Confirmed cases and symptom search distribution exhibit high similarity with high concentration in Midwest and Northeast US, and medium concentration in Western and Southern US. In contrast, the search for LD itself is heavily focused in Northeast and Midwest US with barely any search from Texas and Western regions. The search for IGeneX is found high in areas with high number of actual cases, although the absolute numbers are relatively low.

**Figure 3:**
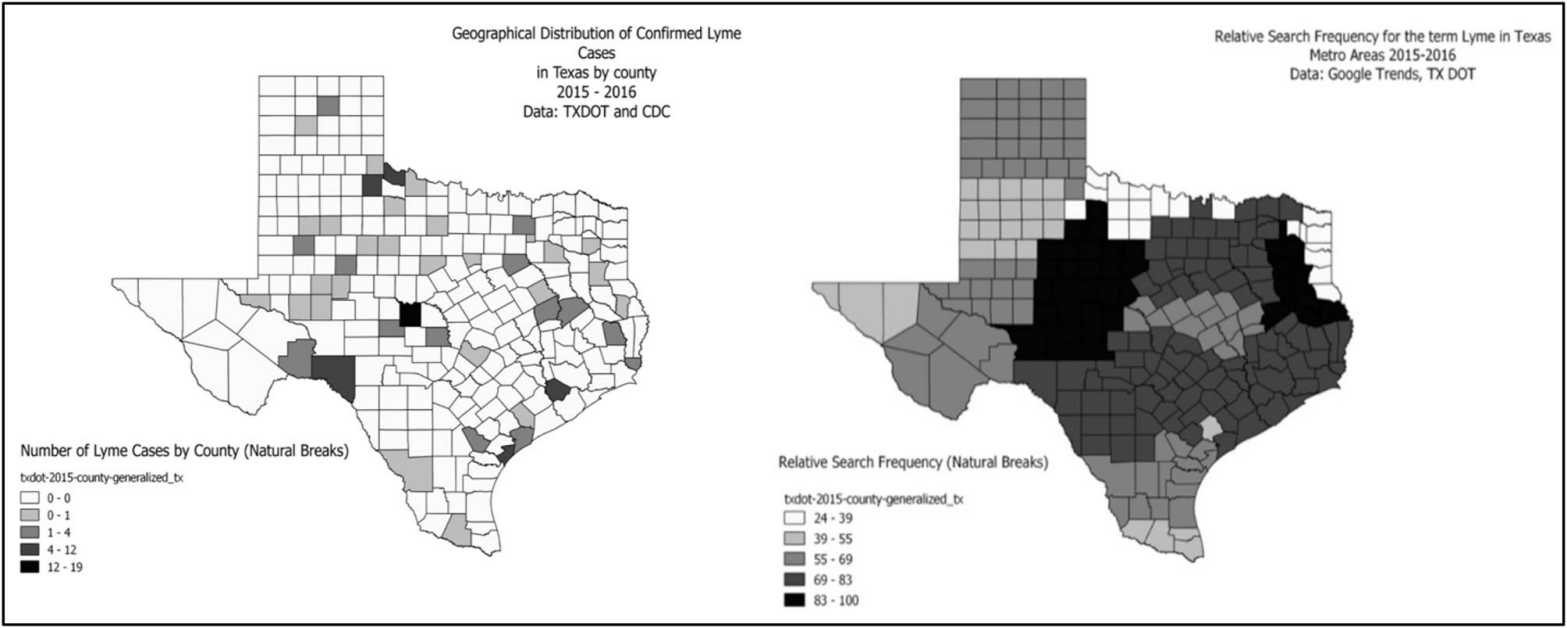
County-level spatial distribution of CDC-confirmed LD cases and Google Trends results for LD in Texas.

There is also a substantial variation even within a state. Figure 3 shows the county-level spatial distribution of CDC-confirmed LD cases and Google Trends results for LD in Texas. A direct comparison seems difficult due to small sample size and metro-scale aggregation of Google Trends outcomes, but it can be considered as a preliminary evidence of spatial mismatch between the confirmed LD cases and perceived LD risks within a state of Texas. In particular, it seems that many people searched for LD in the north and central regions of Texas while no case was confirmed in most of the counties in the regions. In fact, Texas has the very real possibility of confusion on LD given the occurrence of Southern Tick-Associated Rash Illness (STARI) which presents as a rash that is almost indistinguishable from the erythema migrans rash of LD (40). The mismatch found in Texas might pertain to look-alike diseases such as STARI as they are not caused by the same organism or associated with the same degree of illness.

## Discussion

The purpose of this study was to review the usefulness and efficacy of Google Trend data tracking for an infectious disease with non-specific symptoms, which may require unique applications and further investigation. Physicians need to be able to recognize and respond to LD in relatively quick fashion to prevent late-stage symptoms from developing in patients. The CDC confirms that LD has historically been reported in all fifty US states, but under-reporting is a recognized manifestation of disease surveillance. Google Flu Trend (GFT) is widely used with burgeoning acceptance in real-time tracking, but diseases lacking undisputed symptomology present surveillance challenges. This study validates the use of Google Trends for disease surveillance in complex diseases. Unlike studies that use only disease-specific terms, such as “the flu,” this study incorporates symptomatic trend data that points to patients in search of a diagnosis. Unconventional use of Google Trends data to review unconventional lab tests is exploratory, but helpful when potentially combined with big data that can potentially distinguish among search pattern causes, such as limiting auto-immune disease of flu during times of intense searches for lab tests such as IGeneX.

By combining different mapping approaches via temporal and spatial at the state-level, this study is the first to explore patient's *perceived* illness in relation to confirmed cases of a disease. Additionally, this study is the first to analyze not just disease symptoms, but lab testing, and specifically, unconventional lab testing. CDC-confirmed LD and Google Trends results for disease name, symptoms, and IGeneX for the years between 2011 and 2015 indicate traditionally understood and expected seasonal patterns. LD is known to peak in the summer with cases and symptoms developing into fall. Lag time with searches are consistent with Google Trends disease studies, and present as searches corresponding with the disease name or, in the case of LD, “tick bite.” The data validate the notion that patients search the internet prior to disease confirmation. Besides, patients searching for a diagnosis pay out-of-pocket or through physicians willing to order IGeneX tests, indicating that these patients struggle with non-specific, ongoing symptoms. These searches do not indicate LD specifically, but rather groups of patients who remain ill and lack a diagnosis of any disease or condition.

Increased search patterns for LD over 5 years indicate that the true risk for LD may be higher than confirmed cases, or undiagnosed patients with similar symptoms to LD are searching for LD. This study also confirms that symptom searches and confirmed cases of LD follow similar patterns overall, but in the northeast, where ticks and LD are more commonly suspected, searches are concentrated by disease name. These results suggest patients unfamiliar with LD may search by symptoms, rather than disease name. This finding is important, as disease surveillance at present is limited to disease name in most studies. This study indicates that algorithms that include multiple surveillance indicators should be considered in future surveillance studies of disease patterns. State-level data also demonstrated that the search patterns in states with substantially different confirmed cases of LD are similar. These findings also suggest that perceived risks and confirmed cases between the states may be mismatched.

This study has numerous limitations that must be considered in the interpretation of the results. This study does not consider the possibility that Google searches often align with when issues are raised in the media, locally or nationally, even if people do not perceive actual risks and simply want to know more about the issues. In addition, it does not perform any statistical analysis to evaluate any temporal or spatial trend reported here due to the inherent limitation of Google Trends data that are displayed as relative search frequencies compared to the maximum. Also, although we tested a lot of possible variations of search terms in Google Trends analysis, including similar disease names and symptoms, to ensure the robustness of this study, our findings cannot be interpreted as confirmatory but should be considered purely exploratory. In addition, a future study should compare the search trends of multiple diseases with similar symptoms with LD to validate the finding of this study.

This study explores the potential use of Google Trends data for unique patterns of data in diseases with complex diagnostics. The use of specialty labs in particular is not a predictive tool for LD *per se*, but this highly unique application of Google Trends searches does tell a story of patients who remain ill. IGENEX is an expensive test that is not used by mainstream physicians and is not covered by insurance. Patients willing to order their own tests and pay hundreds of dollars for validation of their symptoms points to a group of individuals with non-specific symptoms who seek a diagnosis.

## Data Availability Statement

The datasets generated for this study are available on request to the corresponding author.

## Author Contributions

DK and SM designed the study. QL performed the data construction and analysis. DK and QL interpreted the results. DK and SM wrote the manuscript. All authors discussed the results and contributed to the final manuscript.

## Conflict of Interest

The authors declare that the research was conducted in the absence of any commercial or financial relationships that could be construed as a potential conflict of interest. The handling Editor declared a past co-authorship with one of the author DK.
